# CD47 expression is decreased in hematopoietic progenitor cells in patients with myelofibrosis

**DOI:** 10.1590/1414-431X20187784

**Published:** 2018-12-10

**Authors:** A. Nonino, J.M. Nascimento, C.C. Mascarenhas, J.F. Mazzeu, R.W. Pereira, R.H. Jacomo

**Affiliations:** 1 Programa de Pós-Graduação em Ciências Genômicas e Biotecnologia, Universidade Católica de Brasília, Brasília, DF, Brasil; 2 Unidade de Hematologia e Hemoterapia, Hospital de Base do Distrito Federal, Brasília, DF, Brasil; 3 Faculdade de Medicina, Universidade de Brasília, Brasília, DF, Brasil; 4 Sabin Medicina Diagnóstica, Brasília, DF, Brasil

**Keywords:** Myeloproliferative disorders, Primary myelofibrosis, Hematopoietic stem cells, Neoplastic stem cells, Antigens, CD47

## Abstract

Myelofibrosis (MF) is characterized by increased circulating hematopoietic progenitor cells (HPCs), abnormal cytokine levels, and the survival advantage of neoplastic progenitors over their normal counterparts, which leads to progressive disappearance of polyclonal hematopoiesis. CD47 is a surface glycoprotein with many functions, such as acting as a phagocytosis inhibitor of the expressing cell, that is increased in normal hematopoietic stem and progenitor cells mobilized into the blood and several human cancer-initiating cells, such as in acute myeloid leukemia. We compared CD47 expression in hematopoietic stem and progenitor cells of patients with MF and controls and found it to be decreased in progenitors of MF. Exposure of control HPCs to the cytokines transforming growth factor β and stromal-derived factor 1, which are important regulators of hematopoietic stem cell cycling and are overexpressed in patients with MF, did not modulate CD47 expression.

## Introduction

Myelofibrosis (MF) is a myeloproliferative neoplasm (MPN) that can present as *de novo* (primary) MF (PMF) or as MF after transformation of polycythemia vera (PV) or essential thrombocythemia (ET). MF is characterized by clonal hematopoiesis, bone marrow stromal changes, and myeloid metaplasia, which cause debilitating symptoms, hepatosplenomegaly, ineffective hematopoiesis, and increased risk of morbidity and mortality because of bone marrow failure, thrombotic/hemorrhagic events, and transformation to acute leukemia (1). Patients with MF frequently present with blood showing a leucoerythroblastic picture and an increased number of circulating hematopoietic progenitor cells (HPC) characterized by the expression of CD34 antigen. The increased number of CD34 cells can help distinguish between MF and other MPNs (2).

MF is an inflammatory disease with elevated circulating levels of many cytokines and growth factors, such as transforming growth factor β (TGF-β) and stromal-derived factor 1 (SDF-1) (3–5). TGF-β has been associated with the development of bone marrow fibrosis and is involved, together with SDF-1, in the regulation of quiescence or cycling of hematopoietic stem cells (HSCs) (6). The abnormal expression of these two cytokines and their receptors on MF HSCs can be associated with myeloproliferation and enhanced circulation of myeloid progenitors, and could collaborate in the disappearance of polyclonal HSCs (7).

More than 85% of patients with MF have a mutually exclusive mutation in one of the following three genes: JAK2 (60–65%), MPL (5%), or CAL-R (20–25%). All of these mutations, which are called “driver” mutations, activate the janus kinase-signal transducer and activator of transcription (JAK-STAT) pathway. The type of driver mutation may have prognostic impact (8,9). Independently of the driver mutation, circulating CAL-R protein is increased in patients with MF, it participates in the inflammatory network, and correlates with the aggressiveness of the disease (10). CAL-R induces phagocytosis, is overexpressed on the surface of many human cancer cells, and its prophagocytic signaling is opposed by CD47 (11).

The ubiquitous cell surface glycoprotein CD47 (integrin-associated protein) is an important regulator of integrin function, but it also interacts with other proteins, such as thrombospondins (TSP) and signal regulatory proteins (SIRP). Depending on the type of cell or biological context, ligation of CD47 may result in cell activation or apoptosis. For instance, ligation of CD47 with TSP-1, a glycoprotein derived from megakaryocytes, which is increased in MF and causes activation of TGF-β (12), can induce proliferation of some cancer cells, such as astrocytoma cells, but not of their normal counterparts (13).

By binding to SIRPα, CD47 can function as a marker of self on host cells (14,15). In the macrophage, triggering of phagocytosis of a target cell is based on the balance between positive prophagocytic signals and inhibitory CD47/SIRPα signaling. In hemophagocytic lymphohistiocytosis, a systemic inflammatory disorder characterized by phagocytosis of HSCs, these target cells were found to express reduced levels of CD47 (16).

CD47 is upregulated on circulating HSCs and on several human hematologic and solid cancer-initiating cells (17–19). This can be an advantageous mechanism for neoplastic cells over their normal counterparts, which allows the former to evade phagocytosis by cells of the innate immune system. CD47 expression on leukemic stem cells (LSCs) predicted worse overall survival of patients with acute myeloid leukemia (AML) and anti-CD47 blocking monoclonal antibodies preferentially enabled phagocytosis of AML leukemic HSCs (20).

The objective of this study was to compare the expression of CD47 antigen on the surface of HSCs, HPCs, and lineage-committed cells from patients with MF and controls. We also tested whether the expression of CD47 could be modulated in control CD34-positive cells when exposed to the abnormal concentrations of TGF-β and SDF-1 seen in patients with MF.

## Material and Methods

### Sample collection

The study was approved by Escola Superior de Ciências da Saúde do Distrito Federal Research Ethics Committee. Patients and controls were followed at Hospital de Base do Distrito Federal, Brasilia, Brazil and gave informed consent in accordance with the Declaration of Helsinki (1975, revised in 2000). Peripheral blood samples (n=8) were obtained from patients with MF whose diagnosis had been established according to the 2008 World Health Organization criteria (21) and confirmed by 2016 criteria (22) and that presented with increased circulating CD34-positive cells (more than 10 cells/μL). Control marrow cells (n=4) were obtained from previously treated patients with acute promyelocytic leukemia (APL) who were in complete hematologic remission after the end of maintenance chemotherapy and who had their bone marrow collected as part of routine minimal residual disease monitoring. All controls were found to be in molecular remission. Table 1 shows the main epidemiologic and clinical characteristics of patients and controls.

**Table 1:** Main epidemiologic and clinical characteristics of myelofibrosis patients and controls.

Characteristics	Myelofibrosis patients	Controls
Age, median (range)	66 (38–85)	36.5 (22–56)
Gender (N)		
Male	3	1
Female	5	3
Diagnosis (N)		
PMF fibrotic phase	6	NA
PMF pre-fibrotic	1	
Post-ET MF	1	
Dynamic international prognosis system (N)		NA
Low	2	
Intermediate 1	4	
Intermediate 2	2	
High	0	
Hemoglobin (g/dL), median (range)	13.3 (8.7–14.9)	14.6 (13.9–14.8)
Leukocytes (cells/mm^3^), median (range)	26,800 (8,060–103,000)	5,250 (4,980–5,370)
Circulating blasts (%), range	0–2	0
Circulating CD34+ cells (/mm^3^), median (range)	59.5 (11.2–476)	NA
Platelets (cells/mm^3^), median (range)	254,000 (165,000–381,000)	196,500 (164,000–276,000)
Chemotherapy at time of sample collection (N)		
None	6	NA
Hydroxyurea	2	

MF: myelofibrosis; PMF: primary myelofibrosis; ET: essential thrombocythemia.

### Driver mutation genotyping

All patients had their genotype for JAK-2, V617F, CAL-R, or MPL mutation tested by multiplex ligation-dependent probe amplification assay with P420-X2 MPN Mix-2 (MrC Holland, Netherlands).

### Cell separation and CD34 enrichment

Mononuclear cells (MNCs) were isolated by density gradient centrifugation using Ficoll-Paque (GE Healthcare Bio-Science AB, Sweden). The MNC fraction was then enriched for CD34 cells using a CD34+ immunomagnetic isolation kit (Miltenyi Biotec, USA) according to the manufacturers' instructions. After enrichment, a minimum of 0.5×10^6^ control cells (median 0.84×10^6^ cells) and 1×10^6^ cells from patients with MF (median 1.8×10^6^ cells) were obtained and taken for further assays. CD34 purity for controls and patients with MF ranged from 29 to 68% (median 60%) and 48 to 92% (median 75%), respectively.

### Cryopreservation

After separation and CD34 enrichment, MNC cells were either frozen in isopropanol and kept at −80°C or directly cultured in the presence of cytokines. Before flow cytometry analysis, cryopreserved cells were thawed at 37°C and washed twice in phosphate-buffered saline (PBS).

### Control cell cultures

MNC CD34-enriched control cells were incubated at approximately 5×10^5^ cells/mL in serum-free medium (Stemline II; Sigma-Aldrich, USA), in the presence or absence of TGF-β (5 ng/mL; Sigma-Aldrich) or SDF-1 (0.5 ng/mL; Life Technologies, USA) for 72 h at 5% CO_2_ at 37.5°C.

### Flow cytometry

Multiparameter analysis of purified cells for surface antigen expression was performed by incubating thawed or cultured cells at room temperature for 15 minutes in PBS/1% bovine serum albumin and washed twice before flow cytometric analysis. The monoclonal antibodies were anti-CD34 APC-Cy7 (clone 581; Biolegend, USA), anti-CD38 V450 (clone HIT2; Exbio, Czech Republic), anti-CD90 PE-Cy7 (clone 5E10; Biolegend), anti-CD47 PerCP Cy5.5 (clone CC2C6; Biolegend), PerCP Cy5.5 isotype (clone MOPC-21, Biolegend), and lineage cocktail FITC against CD3, CD14, CD16, CD19, CD20, CD56 (Cat.No.6K01-T050; Exbio). Flow cytometric analysis was performed on a Beckton-Dickson FACS Canto II. A minimum of 2400 events (median 16,500) for CD34 positive cells were obtained in each sample.

**Figure 1:**
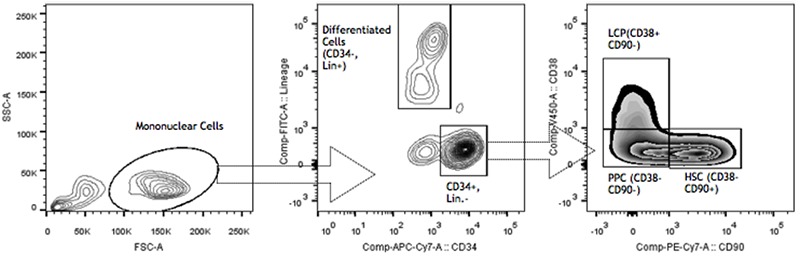
Gating Strategy. LCP: lineage committed progenitors; PPC: pluripotential progenitor cells; HSC: hematopoietic stem cells.

For CD47 expression analysis of non-cultured cells, we defined different cell populations as follows: HSC (CD34+CD38- CD90+Lin-), pluripotential progenitor cells (PPC) (CD34+CD38-CD90-Lin-), lineage-committed progenitors (LCP) (CD34+CD38+Lin-), and differentiated cells (DC) (CD34-Lin+) of patients and controls. Figure 1 shows the gating strategy. For every population, CD47 expression intensity was calculated by subtracting its mean fluorescence intensity (MFI) from the isotype MFI.

### Statistical analysis

Statistics were calculated using Prism 4.0 software (GraphPad Software, USA). Comparison of CD47 expression between MF and control cells and between treated versus non-treated control cells used the Welch-corrected Student's *t*-test. For comparison of expression of different groups of cells from patients with MF or controls, one-way analyses of variance (ANOVA) was used. *Post hoc* analysis of ANOVA data included test for linear trend and correction for multiple comparisons with Bonferroni test. Two-tailed P values <0.05 were considered statistically significant.

## Results

### CD47 expression was reduced in MF CD34-positive cells

**Figure 2:**
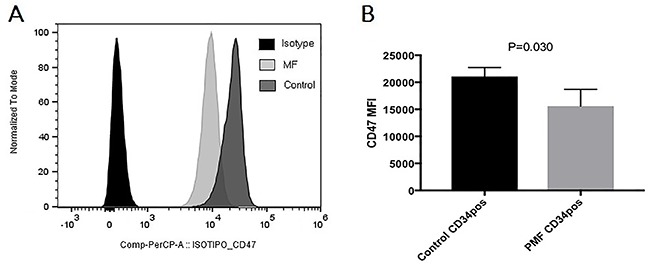
CD47 expression in control *vs* myelofibrosis (MF) CD34-positive (pos) cells. *A*, Histogram of representative experiments; *B*, Comparison of average mean fluorescence intensity (MFI). Data are reported as mean±SD; Welch-corrected Student's *t*-test. PMF: primary myelofibrosis.

We measured CD47 expression in control and MF CD34-positive cells. The mean MFI of control cells was higher than that of MF cells (20181±1520 *vs* 14961±955, P=0.0302) (Figure 2).

**Figure 3:**
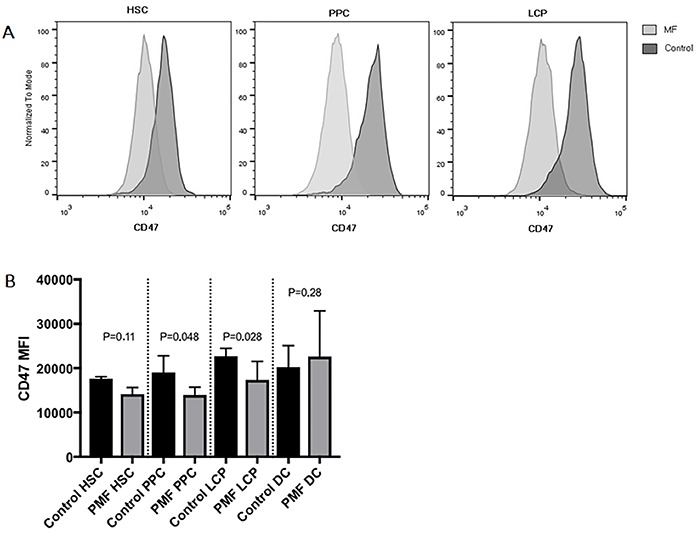
CD47 expression in different cell compartments of control *vs* myelofibrosis (MF) cells. *A*, Histograms of representative experiments; *B*, Comparison of average mean fluorescence intensity (MFI) (Welch-corrected Student's *t*-test). Data are reported as mean±SD. HSC: hematopoietic stem cells; PPC: pluripotential progenitor cells; LCP: lineage committed progenitors; DC: differentiated cells.

When we analyzed each cell compartment, the expression of CD47 was significantly reduced in MF PPCs (CD34+CD38-CD90-Lin-) and LCPs (CD34+CD38+Lin-) (P=0.048 and 0.028, respectively) (Figure 3).

### CD47 expression in CD34+ cells increased with cell differentiation

**Figure 4:**
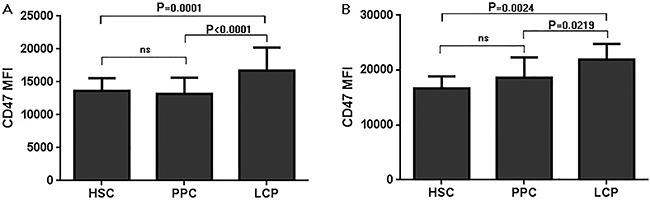
Comparison of CD47 expression among different CD34-positive cell compartments (ANOVA with Bonferroni post-test). Data are reported as mean±SD. *A*, Patients with myelofibrosis; *B*, Controls; MFI: Mean fluorescence intensity; HSC: hematopoietic stem cells; PPC: pluripotential progenitor cells; LCP: lineage committed progenitors; ns: non-significant.

Figure 4 shows that there was a pattern of increasing CD47 expression along with the differentiation of CD34+ cells from HSC and PPC to LCP, for both patients with MF and controls (P values for linear trend between column mean and left-to-right column order: <0.001 and <0.05, respectively).

### Driver mutation did not influence CD47 expression in MF HSC

The distribution of patients according to driver mutation was as follows: four patients were JAK-2 V617-positive, two were CAL-R type 2 (insertion)-positive, one was MPL 515L-positive, and one was triple negative. We compared CD47 expression in JAK2 V617F-positive and -negative patients and CAL-R-positive and -negative patients. There was no significant difference between these groups (P=0.843 for JAK2-positive or -negative and P=0.359 for CAL-R-positive or -negative patients).

No other specific feature of patients with MF was found to influence CD47 expression, including CD34-positive cell frequency in the peripheral blood or hydroxyurea (HU) treatment (P*=*0.25 for HU *vs* non-treated). When the two patients treated with HU were excluded from the comparison with control cells, the difference between patients with MF and controls kept its significance (P*=*0.026).

### Exposure to cytokines did not influence CD47 expression

**Figure 5:**
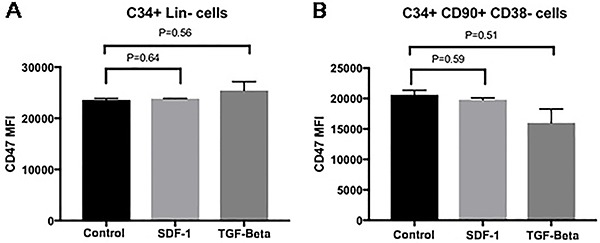
CD47 expression after exposure to SDF-1 and TGF-ß. *A*, Comparison in CD34 positive cells and *B*, Hematopoietic stem cells (Welch-corrected Student's *t*-test). Data are reported as mean±SD. MFI: mean fluorescence intensity.

We analyzed CD47 expression in control CD34-positive and HSCs after 3-day culture in stem cell media exposed to SDF-1 and TGF-β in concentrations similar to those previously described for the serum of patients with MF. These cytokines did not seem to modulate CD47 expression in these experimental conditions (Figure 5).

## Discussion

The progressive exhaustion of normal HSCs and mobilization of HPCs to the peripheral blood are hallmarks of MF. Since mobilized normal HSCs and LSCs of some acute myeloid neoplasms show increased CD47 expression, we hypothesized that this could also be found in progenitor cells from patients with MF.

In this study, we compared the expression of CD47 in different hematopoietic populations from controls (obtained from bone marrow) and patients with MF (obtained from peripheral blood). We found that CD34-positive cells derived from patients with MF have lower CD47 than their normal sessile bone marrow counterparts.

One previous study (17) demonstrated that CD47 is upregulated in AML and blastic phase CML, but not in other myeloproliferative disorders, including PV, post-PV MF, ET, and PMF (n=5). Unlike that report, our study tested CD47 expression not only in total CD34-positive population, but also in different hierarchic hematopoietic populations. We found that there was an increasing pattern for CD47 expression as patients' or controls' HPCs matured to LPC and that patients with MF showed comparatively decreased CD47 expression in PPC (CD34 pos, CD90neg, CD38neg, Lin neg) and LCP (CD34 pos, CD90neg, CD38pos, Lin neg).

As HPCs from patients with MF and controls were submitted *in vivo* to different microenvironment conditions, we tested whether this difference could be due to hematopoietic cell exposure to two of the most pathophysiological important cytokines found elevated in patients with MF serum, SDF-1 and TGF-β. We showed that CD47 expression in control CD34-positive cells or HSCs was not modulated by high concentrations of these cytokines after 72-h culture in stem cell media.

We currently do not know whether our findings have any relevance to the pathophysiology of MF. CD47 is a glycoprotein with multiple roles. Although one of its best characterized functions is inhibiting phagocytosis by macrophages, even this role can be modulated by different protein interactions, as demonstrated in red blood cells (RBC). CD47 can act as a molecular switch controlling RBC phagocytosis because it undergoes structural changes in aged erythrocytes, favoring binding to TSP-1, which is a protein that is abundant in the bone marrow stroma of MF (12). This interaction with TSP-1 changes the “don’t eat me” signal to a phagocytosis-inducing signal (23). Also, despite the description of CD47 expression as an advantageous feature for neoplastic cells, CD47 ligation by monoclonal antibodies can induce apoptosis in many tumor cell lines, such as chronic lymphocytic leukemia cells (24), and binding of CD47 by TSP-1 has also been described to induce cell apoptosis (14).

Our study has some possible limitations that deserve comment. Cells from controls and patients with MF were from different sources because bone marrow aspiration in patients with MF is usually unsuccessful. However, considering previous results showing that resident bone marrow HSCs express less CD47 than circulating cells, the different sources of cells could underestimate the difference between controls and MF cells. It could also be argued that the CD34 cells from patients with MF do not represent solely the neoplastic clone, but a mixture of normal and MF cells. However, unlike other MPNs, the neoplastic clone is predominant in MF hematopoiesis and, again, the admixture of normal and malignant cells could underestimate the difference, instead of causing it. Although the control samples were obtained from previously treated APL patients instead of healthy individuals, they were free of cytotoxic treatment for more than 3 months, were proven not to have minimal residual disease by polymerase chain reaction, and had normal blood counts and, therefore, normal hematopoiesis. Two of the 8 patients with MF were receiving treatment with HU at the time of sample collection, but their average CD47 expression did not differ from those without HU. Also, excluding these 2 patients from the analysis did not affect our findings.

In conclusion, in our experimental conditions, HPCs from patients with MF have reduced CD47 expression compared to control cells. These findings suggest that CD47-related inhibition of phagocytosis of neoplastic cells by macrophages may not play a role in the survival advantage of MF progenitors over their non-clonal counterpart, although this has not been tested.

We believe our results may stimulate further investigation on the possible role of HPCs' CD47-reduced expression on the pathogenesis of MF.
